# Dropped head syndrome secondary to head and neck cancer: Impact on functıonal and body image scale

**DOI:** 10.1016/j.heliyon.2024.e38614

**Published:** 2024-09-29

**Authors:** Songül Keskin Kavak, Engin Eren Kavak

**Affiliations:** a Ankara Dr.Abdurrahman Yurtaslan Training and Research Hospital Department of Physical Therapy and Rehabilitation, Turkey; b Ankara University Medical Faculty, Department of Medical Oncology, Turkey

**Keywords:** Head and neck cancer, Dropped head syndrome, Body image scale, Quality of life, Head and neck lymphoedema

## Abstract

**Objective:**

The primary aim of our prospective study was to assess the impact of Dropped Head Syndrome (DHS), a rare condition in Head and Neck Cancer (HNC) clinics, on patients' functional status and body image. Our secondary aim is to investigate the relationship between head and neck lymphoedema (HNL) and DHS, which will be examined for the first time in the literature.

**Methods:**

We conducted a study involving 47 patients, aged between 18 and 75, who had been diagnosed with HNC, and exhibited clinical symptoms of DHS for at least 12 months. The staging of HNL was assessed using the MD Anderson Cancer Center HNL (MDACC HNL) staging system. We also administered The Total Functional Scale (TFS) which was a subscale of EORTC QLQ-C30 (European Organization for Research and Treatment of Cancer Quality of Life Questionnaire C30) and the Body Image Scale (BIS).

**Results:**

In the study, it was observed that the BIS was significantly lower in patient groups aged 50 and over (p = 0.0495), those with a laryngectomy (p = 0.0002), those who had undergone bilateral neck dissection (p = 0.0291), and particularly in patients with stage 2–3 lymphedema (p < 0.0001). Similarly, it was noted that passive cervical extension limitation had a statistically significant impact on both the BIS (p < 0.0001) and the TFS (p < 0.0001). It was also found that BIS (p < 0.0001) and TFS (p < 0.0001) improved in the late postoperative period (12 months ≤) and this improvement was statistically significant.

**Conclusions:**

In this study, we found statistical relationships between age, laryngectomy, surgery procedures, lymphedema stages, passive cervical extension limitations, total functional score, and BIS. Early diagnosis of DHS allows for supportive care and physiotherapy methods, which can lead to improvement. HNL and DHS should be prevented to improve quality of life and body image and increase survival. Therefore, further research with a much larger patient population is needed.

## 1 Introduction

Head and Neck Cancer (HNC) is a significant global health issue, ranking as the seventh most common cancer worldwide, with more than 660,000 new cases and approximately 325,000 deaths reported annually [1]. The World Health Organization (WHO) employs the International Classification of Diseases (ICD-10) to categorize head and neck cancers based on their anatomical locations. This classification encompasses various regions, including the hypopharynx, oropharynx, nasopharynx, larynx, tongue, gingiva, salivary glands, parotid gland, and nasal cavity [2].

In classical radical neck dissection (RND), which is commonly performed in Head and Neck Cancer (HNC) surgery, several structures, including the sternocleidomastoid muscle, jugular vein, XI^th^ cranial nerve (accessory nerve), cervical lymph nodes, and submandibular gland, are surgically removed [3]. Secondary head and neck lymphedema, which develops due to disruption of normal lymphatic flow in patients who have undergone surgery and radiotherapy for head and neck cancers, is a chronic condition that affects the quality of life [4]. The edema that develops in the mouth and throat due to head and neck lymphoedema (HNL), and the asymmetrical appearance of the neck as a result, causes panic and anxiety in the patient and increases concerns about body image [5].

A rare condition known as dropped head syndrome (DHS) can result from head and neck surgery, postradiotherapy, and HNL.The patient presents with a constellation of symptoms, including HNL, extensor muscle atrophy in the neck, severe weakness of the cervical paraspinal muscles, and marked fibrosis of the anterior flexor muscles resulting in DHS [6]. This syndrome is characterized by the head falling forward and not being able to keep it upright without external support. DHS occurs secondary to various factors, most commonly neuromuscular disorders, cervical spine surgery, surgical treatment for head and neck cancers, and radiation therapy [7].

Although studies have frequently characterised DHS in the context of oncological rehabilitation as a late complication resulting from radiotherapy, there are sporadic rare case reports in the literature describing early-onset cases. This suggests that DHS may not be exclusive to late-stage complications and can, under certain circumstances, manifest earlier during treatment or recovery [8,9].

The primary aim of our study is to evaluate DHS, which is a common condition in HNC clinics and affects the functional status and body image of the patient. Our secondary aim was to examine the relationship between HNL and DHS. The strength of our study is that it is the first study to examine the relationship between HNL and DHS.

## 2 Material and methods

**Fig. 1:**
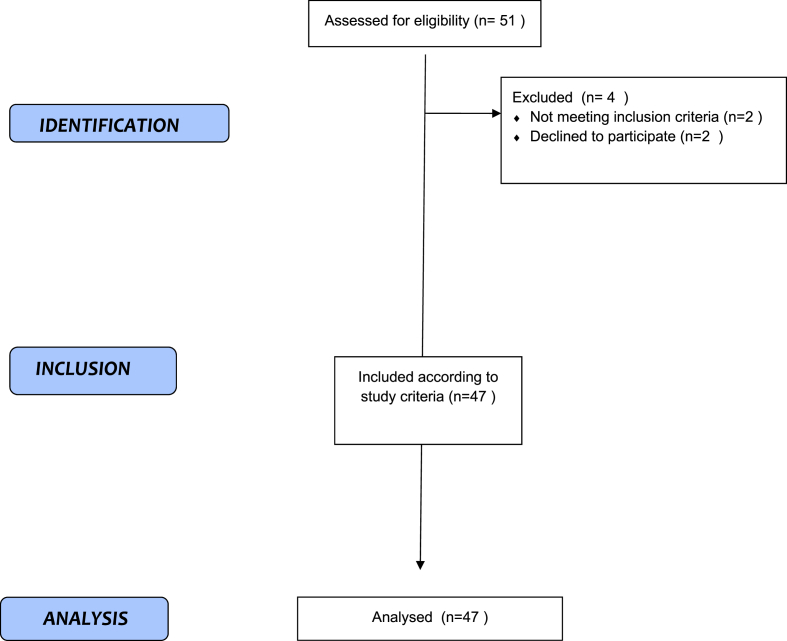
Strobe flow diagram.

The present study is a prospective investigation conducted at the Oncological Rehabilitation Clinic of the Oncology Education and Research Hospital in Turkey, a tertiary healthcare facility. The study was conducted between April and June 2023. The objective was to evaluate 47 patients, ranging in age from 18 to 75 years, who had previously undergone treatment for HNC (see Fig. 1).

### 2.1 Participant characteristics

**Fig. 2:**
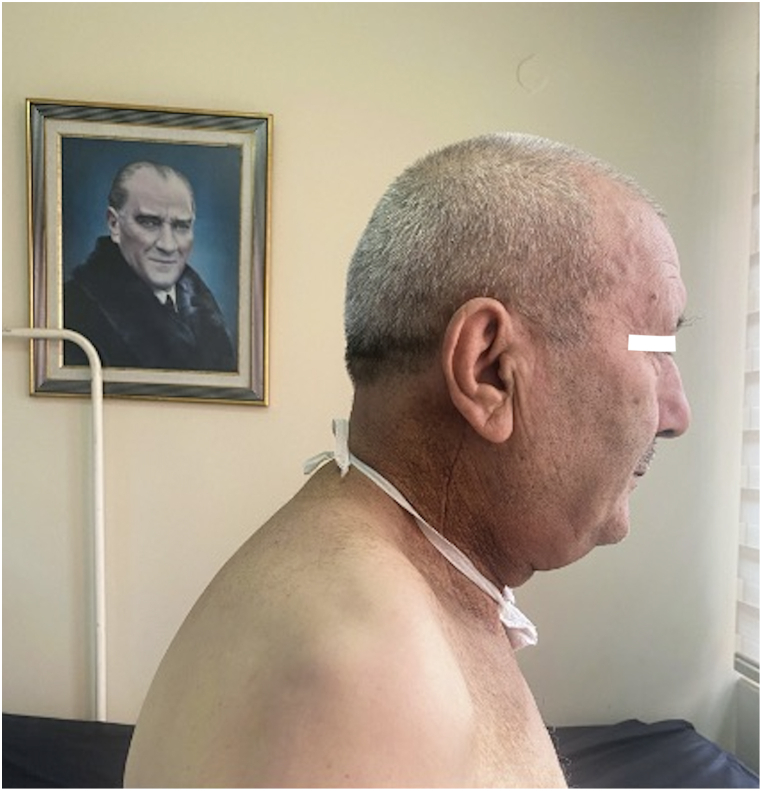
Patient with dropped head syndrome secondary to head and neck cancer.

The inclusion criteria for the study consisted of patients who had clinical manifestations of DHS for at least 12 months, including tenderness and pain on palpation of the cervical paraspinal muscles, limitation of cervical extension, and development of chin-chest deformity (Fig. 2). Patients who had completed treatment for head and neck cancer, including surgery and/or chemoradiotherapy, were included in the study. Exclusion criteria for the study were seen as the most common causes of DHS; neuromuscular diseases, Parkinson's disease, spinal cord injuries, cervical spinal surgeries, and also recurrent disease, distant metastases, deep vein thrombosis, advanced heart failure, severe arterial disease, septic venous inflammation, and patients currently receiving chemoradiotherapy.

Demographic and clinical information of the patients including age, sex, body mass index (BMI) in kg/m^2^, type of surgery (bilateral neck dissection/unilateral neck dissection) and presence of laryngectomy were recorded.

All patients underwent radiation therapy. The administration of radiotherapy was conducted in accordance with the specific characteristics of the primary disease, stage of progression, and performance status of the patient. Following surgery and radiotherapy, all patients developed HNL. Staging of HNL was performed using the MD Anderson Cancer Center HNL (MDACC HNL) staging system. Measurements were performed by the same lymphedema-trained physical medicine and rehabilitation specialist.

### 2.2 Patient evaluation methods

The range of motion of the cervical joints, which had been restricted as a consequence of the patient's DHS, was evaluated through the use of manual goniometry, with the resulting measurements were recorded.

The EORTC QLQ-C30 (European Organization for Research and Treatment of Cancer Quality of Life Questionnaire C30) and the Body Image Scale (BIS) questionnaire, which are specific quality-of-life assessment tools for cancer patients, were administered. The methodology employed for the assessment of patients is delineated in the following paragraphs.

The European Organization for Research and Treatment of Cancer Quality of Life Questionnaire Core 30 (EORTC QLQ-C30) and Total Functional Scale (TFS):

The EORTC QLQ-C30 is a cancer-specific quality-of-life assessment tool that consists of 30 items. It includes five functional scales (physical, role, cognitive, emotional, and social functioning), three symptom scales (fatigue, nausea and vomiting, and pain), a general health status/quality of life scale, and six single items (appetite loss, diarrhea, dyspnea, constipation, insomnia, financial impact).

The EORTC QLQ-C30 assesses all scales and single-item components on a scale of 0–100. In the first 28 questions that measure functional and symptom scales, respondents provide ratings on a four-point Likert scale, ranging from “not at all” (1 point) to “a little” (2 points), “quite a bit” (3 points), and “very much” (4 points). The 29th question requests that the respondent evaluate their overall health on a scale from 1 to 7, with 1 indicating “very poor” and 7 indicating “excellent.” The 30th question assesses the respondent's overall quality of life. Higher scores for Total functional Scores, and general health scores indicate better health and quality of life, while higher scores on symptom scores indicate a greater presence of symptoms and problems [10] (Table 1). The validity and reliability of the EORTC QLO-C30 have been demonstrated in a Turkish population [11].

**Table 1:** EORTC QLQ-C30.

1. Do you have any trouble doing strenuous activities, like carrying a heavy shopping bag or a suitcase?
2. Do you have any trouble taking a long walk?
3. Do you have any trouble taking a short walk outside of the house?
4. Do you need to stay in bed or a chair during the day?
5. Do you need help with eating, dressing, washing yourself or using the toilet?
**During the past week:**
6. Were you limited in doing either your work or other daily activities?
7. Were you limited in pursuing your hobbies or other leisure time activities?
8. Were you short of breath?
9. Have you had pain?
10. Did you need to rest?
11. Have you had trouble sleeping?
12. Have you felt weak?
13. Have you lacked appetite?
14. Have you felt nauseated?
15. Have you vomited?
16. Have you been constipated?
17. Have you had diarrhea?
18. Were you tired?
19. Did pain interfere with your daily activities?
20. Have you had difficulty in concentrating on things, like reading a newspaper or watching television?
21. Did you feel tense?
21. Did you feel tense?
22. Did you worry?
23. Did you feel irritable?
24. Did you feel depressed?
25. Have you had difficulty remembering things?
26. Has your physical condition or medical treatment interfered with your family life?
27. Has your physical condition or medical treatment interfered with your social activities?
28. Has your physical condition or medical treatment caused you financial difficulties?
**For the following questions please circle the number between 1 and 7 that best applies to you**
29. How would you rate your overall health during the past week?
30. How would you rate your overall quality of life during the past week?

The responses to questions 1–28 are scored on a four-point scale, with 1 indicating “Not at all,” 2 indicating “A little,” 3 indicating “Quite a bit,” and 4 indicating “Very much.” The responses to questions 29 and 30 will be scored on a scale from 1 (very poor) to 7 (excellent).

In our study, we evaluated the correlation between DHS and the total functional score (TFS).

To calculate the TFS, the patient's total score from 15 questions is divided by the total number of questions (15) to calculate the raw score [1]. Utilizing these values, the following equation was employed to calculate TFS: {1-(RS-1)/range}x100 [10].

#### 2.2.1 Body image scale (BIS)

The BIS consists of 40 items, each of which describes an organ or part of the body (such as arms, legs, face) or a function (e.g., sexual activity level). These are expressed as “I dislike it a lot,” “I dislike it,” “Undecided,” “I like it,” and “I like it a lot.” A score of 1–5 is given for each item. The total score on the scale ranges from 40 to 200, with a higher score indicating a higher level of satisfaction [12] (Table 2). The scale was adapted into Turkish and validity/reliability studies were conducted by Hovardaoğlu [13].

**Table 2:** Body image scale.

1. My hair	11. My energy levels	21. The width of my shoulders	31. My feet
2. The color of my face	12. My back	22. My arms	32. My sleep pattern
3. My appetite	13. My ears	23. My chest	33. My voice
4. My hands	14. My head	24. The shape of my eyes	34. My overall health
5. Distribution of body hair	15. My chin	25. My digestive system	35. My sexual activities
6. My nose	16. My body build	26. My hips	36. My knees
7. My physical appearance	17. My profile	27. My immunity to disease	37. The posture of my body
8. My urination frequency	18. My height	28. My legs	38. The shape of my face
9. My muscle strength	19. My sensory sharpness	29. The shape of my teeth	39. My weight
10. My waist	20. My pain tolerance	30. My sexual strength	40. My sexual organs

Each of an organ or part of the body or a function. These are expressed as “I dislike it a lot,” “I dislike it,” “Undecided,” “I like it,” and “I like it a lot.” A score of 1–5 is given for each item.

The MD Anderson Cancer Center Head and Neck Lymphedema (MDACC HNL) staging:

The MD Anderson Cancer Center HNL (MDACC HNL) classification system is used to assess the severity of lymphedema in HNC patients. This system classifies the stages of lymphedema as follows.

• Stage 0: No visible edema, but the patient experiences a sensation of heaviness.
• Stage 1: Soft, reversible edema is present.
• Stage 2: Hard, irreversible edema exists without tissue changes.
• Stage 3: Irreversible tissue changes, such as hyperkeratosis and papillomatosis, are observed [14].

### 2.3 Statistical analysis

General descriptive statistics were presented as mean ± standard deviation for continuous variables and as median (minimum-maximum) when appropriate. Categorical data were expressed as counts and percentages. Independent samples t-tests and ANOVA One Way were used for comparing groups (parametric variables), and Pearson Spearman correlations were used to examine associations of the two outcome scores with continuously coded demographic/clinical variables. A p-value of <0.05 was considered statistically significant.

### 2.4 Power analysis

According to the post-hoc power analysis performed using the G∗Power 3.1.9.2 program, The G Power analysis used a pre-determined effect size (Cohen's d) value of 0.5. The alpha level (Type I error) was set at 0.05, and the power level was set at 0.95. Given that a balanced sample size was used in the study, 45 patients were found. The actual power was found to be 0.951 with a 5 % margin of error. To provide a statistically robust analysis for the effect size, alpha level, and power level determined in our study, a total of 47 patients were included.

## 3 Results

The sociodemographic and clinical characteristics of the 47 patients included in the study are summarized in Table 3.

**Table 3:** The sociodemographic and clinical characteristics of the patients n [%], mean ± SD.).

Characteristic	DHS group (n = 47)
Age (years)	55.12 ± 8.08 (37–72)
Female/Male	12 (25.53)/35 (74.46)
BMI (kg/m^2^)	18.61 ± 2.17 (16–24)
Primer	
Larynx	19 (40.4)
Oral cavity	13 (27.6)
Nasopharynx	8(17.0)
Oropharynx	3(6.3)
Hypopharynx	2 (4.2)
Salivary gland	2(4.2)
Stage_	
I	6(12.7)
II	14(29.7)
III	20(42.5)
IVA	7(14.8)
ECOG PS	
0	27(57.4)
1	15(31.9)
2≤	5(10.6)
Treatment	
Adjuvant RT	5(10.6)
ACC	17(36.1)
IC + CC	14(29.7)
CC	11(23.4)
Laryngectomy	12 (25.53)
Surgery	
Unilateral neck dissection	18 (38.29)
Bilateral neck dissection	29 (61.70)
Time after Treatment (months)	18.17 ± 7.88 (12–48)
Head and Neck Lymphedema (HNL) stage	
(MDACC HNL)	
Stage 1 HNL	13 (27.65)
Stage 2 HNL	22 (46.48)
Stage 3 HNL	12 (25.53)
Total Functional Score (EORTC QLQ-C30) mean ± S.D. (min-max)	48.05 ± 29.88 (14.00–94.00)
Body Image Scale, mean ± S.D. (min-max))	142.80 ± 22.98 (110.00–178.00)
Passive Cervical Extension Limitation, mean ± S.D. (min-max)	25.93 ± 8.29 (8.00–38.00)

Abbreviations:S.D.: Standart Deviation, Min-Max: minimum-maximum, BMI: Body Mass Index, TFS: Total Functional Score, BIS: Body Image Scale; RT: Radiotherapy; ACC:Adjuvant Concurrent Chemoradiotherapy; IC: Induction Chemotherapy; CC: Concurrent Chemoradiotherapy.

According to the comparison of TFS and BIS between <50 and ≥ 50 Age groups, the average TFS for the 12 patients under the age of 50 was 61.14 ± 26.67, and their BIS was 154.00 ± 16.94. For the 35 patients aged 50 or older, the TFS was 43.57 ± 29.94, and the BIS was 138.97 ± 23.71. Although no statistically significant difference was observed in the TFS between the two populations (1.7999, p = 0.0786), a significant statistical difference was evident in the BIS (t = 2.0189, p = 0.0495).

When the BIS was evaluated according to gender in HNC patients with secondary dropped head syndrome, the BIS for the 12 women was 147 ± 26.11, and for the 35 men in the study was found to be 141.37 ± 22.04. There was no statistically significant difference in terms of BIS between the female and male patient groups (t = 0.7283, p = 0.4702). The average TFS in women was 56.50 ± 30.10, while in men, 45.16 ± 29.68. There was no statistically significant difference in the TFS between females and males (t = 0.7283, p = 0.4702).

For patients with a laryngectomy, the average BIS was 123.00 ± 10.10, while for those without a laryngectomy, the average BIS was 149.60 ± 22.26. These scores were found to be statistically significant (t = −3.9782, p = 0.0002). When evaluating the patient's TFS, those with a laryngectomy had an average TFS of 26.58 ± 15.76, while those without a laryngectomy had an average TFS of 55.42 ± 30.15. There was a statistically significant difference in TFS between patients with and without a laryngectomy (t = −3.1525, p = 0.0029).

For patients who underwent unilateral neck dissection, the average BIS was 152.00 ± 23.47, while for those who underwent bilateral neck dissection, the BIS was 137.10 ± 21.10. These scores were also found to be statistically significant (t = 2.2536, p = 0.0291). When comparing the TFS in the same group of patients, those who underwent unilateral neck dissection had an average TFS of 62.20 ± 30.26, while those who underwent bilateral neck dissection had an average TFS of 39.27 ± 26.50. There was a statistically significant difference in TFS between patients who underwent unilateral and bilateral neck dissection (t = 2.7307, p = 0.009).

Comparing the different stages of HNL concerning TFS, a significant relationship emerged. Patients in Stage 3 HNL exhibited the lowest TFS (24.00 ± 18.50) among the groups. This was followed by patients in Stage 2 HNL (40.78 ± 25.04), while those in Stage 1 HNL had the highest mean TFS (82.56 ± 6.71). Statistical analysis using a one-way ANOVA test demonstrated a significant decrease in TFS as lymphedema severity advanced (29.6825, p < 0.001). The evaluation of BIS among patients with head and neck cancer-related DHS revealed the following findings: In patients with Stage 1 HNL, the average BIS was 170.92 ± 6.76. For patients with Stage 2 HNL; 136.18 ± 17.33. Among those with Stage 3 HNL, the BIS was 124.50 ± 14.06. A statistically significant decrease in the BIS was observed as the severity of lymphedema increased (37.1586, p < 0.001) (Table 4).

**Table 4:** The impact of sociodemographic and clinical data on total functional score and body image.

Characteristic	N	TFS (mean ± S.D.)	BIS(mean ± S.D.)
Age <50 Age ≥50	12 35	61.14 ± 26.67	154.00 ± 16.94
		43.57 ± 29.94	138.97 ± 23.71
		t = 1.7999, p = 0.0786^a^	t = 2.0189, p = 0.0495^a^
F M	12 35	56.50 ± 30.10	147 ± 26.11
		45.16 ± 29.68	141.37 ± 22.04
		t = 1.1389, p = 0.2608^a^	t = 0.7283, p = 0.4702^a^
Laryngectomy + Laryngectomy -	12 35	26.58 ± 15.76	123.00 ± 10.10
		55.42 ± 30.15	149.60 ± 22.26
		t = −3.1525, p = 0.0029^a^	t = −3.9782, p = 0.0002^a^
Unilateral neck dissection Bilateral neck dissection	18 29	62.20 ± 30.26	152.00 ± 23.47
		39.27 ± 26.50	137.10 ± 21.10
		t = 2.7307, p = 0.009^a^	t = 2.2536, p = 0.0291^a^
Stage 1 HNL Stage 2 HNL Stage 3 HNL	13 22 12	82.56 ± 6.71	170.92 ± 6.76
		40.78 ± 25.04	136.18 ± 17.33
		24.00 ± 18.50	124.50 ± 14.06
		F = 29.6825, p < 0.001^b^	F = 37.1586, p < 0.001^b^

Abbreviations:S.D.: Standart Deviation, Min-Max: minimum-maximum, F: Female, M: Male, TFS: Total Functional Score, BIS: Body Image Scale.

a Student T-test.

b One way ANOVA.

A statistically significant correlation was observed between passive cervical extension limitation and BIS. This correlation indicated that patients with more severe cervical extension limitations due to DHS experienced a deterioration in their body image (r = −0.6362, p < 0.0001). When evaluating the TFS, a similar statistically significant relationship was observed (r = −0.5922, p < 0.0001).

When evaluating the impact of body mass index (BMI) on BIS, no statistically significant relationship was found (r = 0.2136, p = 0.2989). Similarly, BMI did not have a significant effect on the TFS (r = 0.2051, p = 0.2989).

The relationship between the postoperative period and the BIS was examined, and a statistically significant relationship was observed (r = 0.5732, p < 0.0001). Similarly, there was a correlation with the TFS (r = 0.5603, p < 0.0001). (Table 5). Body image scores of the patients were higher in the early postoperative period.

**Table 5:** Correlation between BIS and TFS and other factors.

	BIS	TFS
Pasive Cervical Extension limitation	r = −0.6362, p < 0.0001	r = −0.5922, p < 0.0001
Body Mass Index	r = 0.2136, p = 0.2989	r = 0.2051, p = 0.2989
Time after surgery	r = 0.5732, p < 0.0001	r = 0.5603, p < 0.0001

Pearson Spearman correlation.

Abbreviations:BMD: Bone mineral density BMI: Body mass index, TFS: Total Functional Score, BIS: Body Image Scale.

A statistically strong and significant relationship was observed between the TFS and the BIS (r = 0.9369, p < 0.0001).

## 4 Discussion

The present study revealed statistically significant correlations between age, laryngectomy, surgical procedures (unilateral/bilateral neck dissection), lymphedema stages, passive cervical extension limitations, time after surgery, and total functional score (EORTC QLQ-C30) with the body image scale.

DHS is characterized by limitations in cervical passive range of motion (PROM), chin-chest deformities, palpable pain in cervical paraspinal muscles, weakness in cervical extensor muscles, and, in advanced stages, severe kyphosis, myelopathy, walking, and coordination problems. In the diagnosis, electromyographic findings are often within the normal range, and in the early stages, conservative methods can be an effective course of treatment [15]. The etiology of DHS is most commonly associated with neuromuscular diseases, Parkinson's disease, spinal cord injuries, cervical spinal surgeries, and, less frequently, head and HNC surgery and post-radiation therapy [16,17]. In addition to the aforementioned diseases, we employed the exclusion criteria of recurrent disease, distant metastases, deep vein thrombosis, advanced heart failure, severe arterial disease, and septic venous inflammation in our study. These conditions may impact the quality of life [18,19]in accordance with our study method, rather than the DHS.

In a cross-sectional investigation that included patients treated up to 14 years earlier, it was found that post-surgery, younger patients undergoing HNC surgery exhibited worse body image compared to the older patient group. Specifically, 25 % of the younger patients reported concerns about their appearance, and 16 % experienced higher expectations of social isolation post-surgery [20]. In our study, body image was more negatively affected in patients over 50 years of age. To reduce the frequency of such problems, especially in elderly patients, if radiotherapy is not beneficial for survival, it may be considered to limit its use.

A small longitudinal pilot study revealed that women who have undergone HNC surgery are more likely to experience poor body image as a result of depression, anxiety, and social isolation [21]. In contrast with the findings of previous studies, our results demonstrated no statistically significant difference in quality of life and body image scores between male and female patient groups. This may be attributed to the relatively low proportion of female patients, who constituted 25.53 % of the total patient population (12 women).

The findings of our study align with those of a previous investigation, indicating that individuals who have undergone laryngectomy tend to exhibit diminished body image [22]. In evaluating the impact of this variable on the quality of life with increased body image stress, it was observed that there was a negative impact on the TFS, which reached statistical significance.

In a study conducted by Chen and colleagues, it was reported that surgeries involving a larger area of excision, such as facial reconstruction and oral excision, had a greater impact on patients' body image than those who underwent less extensive surgery [23].It is well documented that the extent of surgical intervention can have a significant impact on a patient's physical appearance and body image [24]. Our findings align with existing literature, indicating that patients who underwent bilateral neck dissection exhibited a more pronounced adverse impact on their body image.

A retrospective study revealed that patients who underwent classical radical neck dissection (RND) exhibited a restricted cervical joint range of motion in 65.4 % of cases at least six months post-procedure. Among patients who underwent unilateral or bilateral neck dissection, 84 % demonstrated limitations in cervical rotation, while 79 % experienced restriction in lateral flexion. These rates were consistent regardless of the side of the neck that was operated on [25]. In another study that focused on overlooked symptoms following RND, it was found that restricted cervical ROM, reported at a rate of 60 %, was the most frequently missed symptom [26]. Our study is in line with the literature, and it supports the presence of DHS in all patients. We found an extension limitation in the passive cervical joint range of motion, with an average of 25.93 ± 8.29 (min 8.00- max 38.00).

Body image is a multidimensional construct that encompasses an individual's perceptions, feelings, and thoughts about the overall appearance, function, and physical competence of their body. Negative changes in body shape following procedures such as cancer surgery can have a detrimental effect on a patient's body image. Previous studies have shown that this negative body image is associated with poorer quality of life in cancer patients [[27], [28], [29]]. Consistent with the literature, our study found that as BIS worsened in HNC patients with DHS, their TFS also worsened.

Postoperative lymphoedema increases dysphagia, weight loss, malnutrition, social isolation, and physical limitations, which in turn affect body image (27). The findings of our study indicate that patients with more severe HNL exhibited a poorer body image.

In the period following cancer surgery, the increased physical, psychological, social, and economic problems associated with surgery and chemoradiotherapy in the first three to six months have a negative impact on quality of life and body image [21,30]. In our study, all patients were assessed >12 months after surgery.

The limitations of this study include the small sample size, the unknown level of education of the patients, and, consequently, the potential for confounding factors to affect the results. However, this research article represents the largest patient group in the literature for HNC patients with dropped head syndrome. Therefore, the study results should be interpreted with caution.

## 5 Conclusion

The findings of this study indicate that body image and activities of daily living are adversely affected in HNC patients who undergo laryngectomy and uni-bilateral neck dissection and subsequently develop HNL-related DHS. These results were found to be statistically significant. The DHS is characterized by the inability to hold the head upright. If undiagnosed, it can cause irreversible problems such as difficulty swallowing, respiratory distress, anxiety, and depression. Early diagnosis of DHS allows for conservative supportive care and physiotherapy methods, which can lead to improvement. Efforts to enhance body image in HNC survivors can simultaneously improve their functional status. To improve the quality of life, body ımage, and functional state of head and neck cancer survivors, prevention of complications such as HNL and DHS will prolong survival. In light of the aforementioned factors, our study can be considered a valuable reference point for future research in this field. Further research is required with a much larger patient population to gain a more comprehensive understanding of the subject matter.

## Funding

This research did not receive any specific grant from funding agencies in the public, commercial, or not-for-profit sectors.

## Availability of data and materials

Data will be provided upon request.

## Ethical approval

The study was approved by the Ankara Dr. Abdurrahman Yurtaslan Ankara Oncology Training and Research Hospital Ethics Committee on May 10, 2023, with approval number 2023-04/179. Informed consent forms were obtained from the patients.

## Informed patient consent

Complete written informed consent was obtained from the patients for the publication of this study.

## CRediT authorship contribution statement

**Songül Keskin Kavak:** Writing – review & editing, Writing – original draft, Visualization, Validation, Supervision, Software, Resources, Project administration, Methodology, Investigation, Funding acquisition, Formal analysis, Data curation, Conceptualization. **Engin Eren Kavak:** Writing – review & editing, Writing – original draft, Visualization, Validation, Supervision, Software, Resources.

## Declaration of competing interest

The authors declare that they have no known competing financial interests or personal relationships that could have appeared to influence the work reported in this paper.
